# PVT1 inhibition stimulates anti-tumor immunity, prevents metastasis, and depletes cancer stem cells in squamous cell carcinoma

**DOI:** 10.1038/s41419-023-05710-6

**Published:** 2023-03-09

**Authors:** Zhen Qin, Wenbo Zhang, Shuo Liu, Yujia Wang, Xin Peng, Lingfei Jia

**Affiliations:** 1 Department of Oral and Maxillofacial Surgery, Peking University School and Hospital of Stomatology, Beijing, 100081 China; 2 Department of Central Laboratory, Peking University School and Hospital of Stomatology, Beijing, 100081 China; 3 National Center for Stomatology & National Clinical Research Center for Oral Diseases & National Engineering Laboratory for Digital and Material Technology of Stomatology, Beijing, 100081 China

**Keywords:** Head and neck cancer, Immune evasion

## Abstract

Cancer stem cells (CSCs) cause tumor metastasis and immune evasion by as-yet-unknown molecular mechanisms. In the present study, we identify a long noncoding RNA (lncRNA), termed *PVT1*, which is highly expressed in CSCs and correlated closely with lymph node metastasis of head and neck squamous cell carcinoma (HNSCC). PVT1 inhibition eliminates CSCs, prevents metastasis, and stimulates anti-tumor immunity, while inhibiting HNSCC growth. Moreover, PVT1 inhibition promotes the infiltration of CD8^+^ T cells into the tumor microenvironment, thereby enhancing immunotherapy by PD1 blockade. Mechanistically, PVT1 inhibition stimulates the DNA damage response, which induces CD8^+^ T cell-recruiting chemokines, while preventing CSCs and metastasis via regulating the miR-375/YAP1 axis. In conclusion, targeting PVT1 might potentiate the elimination of CSCs via immune checkpoint blockade, prevent metastasis, and inhibit HNSCC growth.

## Introduction

Squamous cell carcinoma accounts for over 90% of all head and neck malignancies and often involves cervical lymph node metastasis [1]. Conventional treatments, including chemotherapy for patients with early-stage disease, have shown curative potential, while recurrent and metastatic diseases remain incurable [2]. Recently, programmed cell death protein 1 (PD1) blockade immunotherapy combined with chemotherapy received approval for the first-line of treatment for recurrent and metastatic head and neck squamous cell carcinoma (HNSCC). Unfortunately, a relatively low objective response rate and short median response duration were observed, indicating the resistance of HNSCC to PD1 blockade [3]. Our recent study used in vivo lineage tracing and genetic approaches to show that cancer stem cells (CSCs) have vital functions in immunotherapy resistance and recurrence/metastasis of HNSCC. CSCs could be targeted to potentiate immunotherapy and reduce tumor recurrence and metastasis [4]. However, the underlying molecular pathways controlling CSCs and their related immune responses remain unknown.

Two major noncoding RNAs (ncRNAs), long noncoding RNAs (lncRNAs) and circular RNAs (circRNAs), were reported to be involved in many molecular pathways, driving specific cell fates and biological responses [5]. Recently, we investigated the roles of circRNAs in regulating tumor stemness and immune evasion. The results showed that in HNSCC, increased levels of circRNA FAT1 regulated the positive association between immune evasion and cancer stemness by promoting the activation of STAT3 [6]. By contrast, lncRNAs are also known to play key roles in tumor recurrence and metastasis, and their aberrant expression is closely associated with HNSCC prognosis [7]. A deeper understanding of the complex interactions coordinated by lncRNAs in metastasis and immune evasion of CSCs would provide a unique opportunity to design better therapeutic interventions.

In this study, we identified that lncRNA *PVT1* regulates cancer stemness, metastasis, and anti-tumor immunity by controlling the DNA damage response and the miR-375/YAP1 axis. We used a chemically-induced Bmi1^CreER^; Rosa^tdTomato^ mouse HNSCC model to determine if *PVT1* could be developed as the target for CSC treatment. This model simulates the development and metastasis of human HNSCC, permitting us to carry out in vivo lineage tracing of Bmi1^+^ CSCs in an immunocompetent tumor immune microenvironment [4, 8]. Targeting PVT1 not only eliminated CSCs, prevented metastasis, and promoted CD8^+^ T cell intratumor infiltration but also inhibited HNSCC growth when combined with PD1 blockade.

## Results

### PVT1 is highly expressed in CSCs and correlates with metastasis

**Fig. 1:**
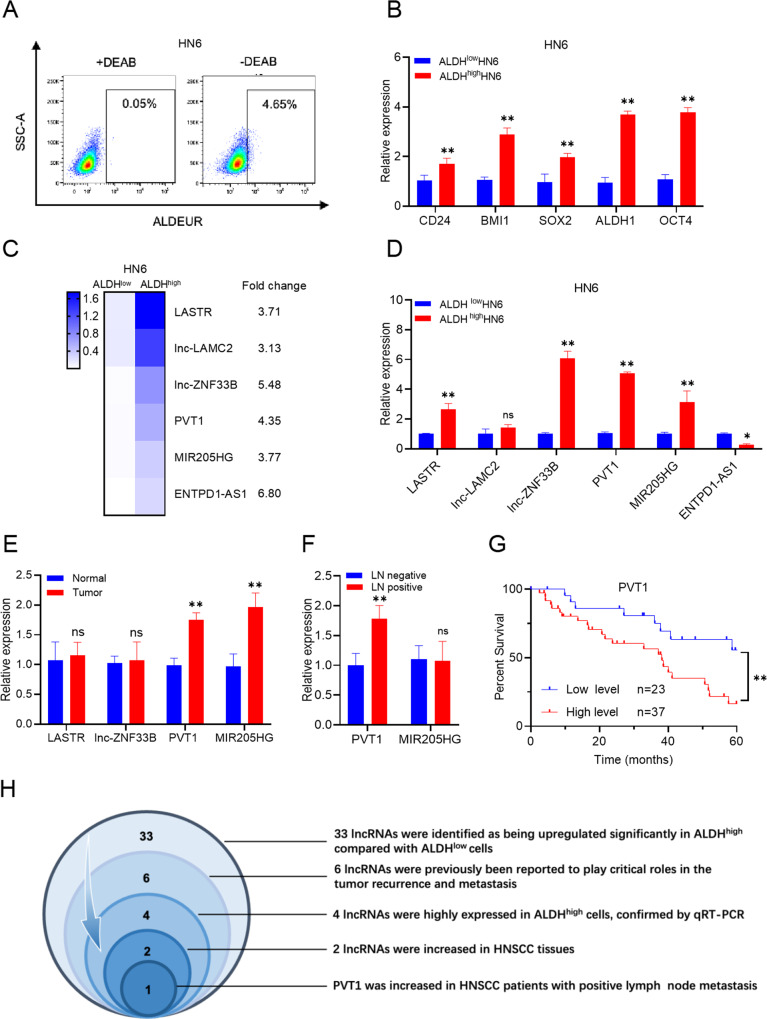
PVT1 is highly expressed in CSCs and correlates with metastasis. **A** ALDH^high^ CSC-like cells and ALDH^low^ non-CSC-like cells were identified among HN6 cells employing fluorescence-activated cell sorting (FACS). An ALDEFLUOR^TM^ kit (ALDEUR) was used to sort ALDH^high^ CSC-like cells as per the manufacturer’s protocol. A specific inhibitor of ALDH, diethylaminobenzaldehyde (DEAB), was used to control for background fluorescence. **B** qRT-PCR analysis of HNSCC CSC-characteristic genes in ALDH^high^ CSCs and ALDH^low^ non-CSCs. The expression levels of *CD24*, *BMI1*, *SOX2*, *ALDH1*, and *OCT4* were normalized to that of *GAPDH* using the 2^−ΔΔCt^ method. Data are shown as the mean ± SD. **p < 0.01 using an unpaired Student’s t-test. **C** RNA-sequencing data heatmap showing the expression of six lncRNAs candidates with known roles in tumor metastasis and recurrence. Fold changes are shown on the right. **D** qRT-PCR assessment of six lncRNAs in ALDH^high^ CSCs and ALDH^low^ non-CSCs. Data are shown as the mean ± SD. ns, *p* > 0^.^05, *p < 0.05, and **p < 0.01 using an unpaired Student’s t-test. **E** qRT-PCR assessment of four lncRNAs in human HNSCC and adjacent normal tissues. Data are shown as the mean ± SD (*n* = 30). ns, *p* > 0.05 and **p < 0.01 using a paired Student’s t-test. **F** qRT-PCR assessment of two lncRNAs in HNSCC tissues with lymph node metastases (LN positive) or without lymph node metastases (LN negative). Data are shown as the mean ± SD (*n* = 20). ns, *p* > 0.05 and **p < 0.01 using a paired Student’s t-test. **G** Kaplan-Meier analysis of the relationship between overall survival and PVT1 expression levels in HNSCC patients (*n* = 60). **p < 0.01 using the Log-Rank test. **H** Schematic of the process of lncRNA screening in HNSCC.

High aldehyde dehydrogenase (ALDH) activity has been successfully used to identify CSCs in HNSCC [9, 10]. ALDH activity can be used to isolate CSCs from human HNSCC cell lines [6, 8, 11]. We used an ALDEFLUOR^TM^ kit to sort ALDH^high^ CSC-like cells and ALDH^low^ non-CSC-like cells from HNSCC cell lines (Fig. 1A). Quantitative real-time reverse transcription-polymerase chain reaction (qRT-PCR) and Western blot showed that the expression of *CD24*, *BMI1*, *SOX2*, *ALDH1*, and *OCT4*, which are well-known genes associated with HNSCC CSC-characters [12], were increased in the ALDH^high^ CSC-like cells (Fig. 1B and Supplemental Fig. S1A). To explore the specific lncRNAs that might mediate the immune evasion of CSCs, RNA sequencing (RNA-seq) data from ALDH^high^ CSCs and ALDH^low^ non-CSCs were analyzed. Differentially expressed lncRNAs between the two groups were identified using cutoffs of a fold change >3.0 and P < 0.01. Thirty three lncRNAs were identified as being upregulated significantly in ALDH^high^ compared with ALDH^low^ cells (Supplemental Fig. S2). Among them, six lncRNAs (*LASTR*, *lnc-LAMC2*, *lnc-ZNF33B*, *PVT1*, *MIR205HG*, and *ENTPD1-AS1*) were previously reported to play critical roles in the tumor recurrence and metastasis [13–17] (Fig. 1C). Then, qRT-PCR confirmed that four of these six lncRNAs were highly expressed in ALDH^high^ cells (Fig. 1D). The expression levels of these four lncRNAs were further examined in 30 samples from cases of HNSCC and their corresponding adjacent normal tissues. This analysis demonstrated that two lncRNAs, *PVT1* and *MIR205HG*, were increased in HNSCC, implying that they might function as oncogenes in HNSCC development (Fig. 1E).

To explore which lncRNA is more closely related with HNSCC metastasis, a case-control study was designed to exclude the influence of certain clinical factors that might influence lymph node metastasis and patient prognosis. PVT1 and MIR205HG expression levels were examined in 20 patients with lymph node-negative HNSCC (LN negative group) compared with that in 20 patients with lymph node-positive HNSCC (LN positive group) who were matched for treatment modality, TNM stage, pathological differentiation, location of the primary carcinoma, sex, and age. The clinicopathological information for these 40 patients with HNSCC were the same as we described previously [6]. We found that PVT1 expression was higher in the LN positive group than in the LN negative group (Fig. 1F). Moreover, the prognosis of patients with HNSCC showing high PVT1 expression was poor (Fig. 1G). Therefore, we chose *PVT1* for further investigation (Fig. 1H).

### Knockdown of PVT1 inhibits HNSCC proliferation and invasion

**Fig. 2:**
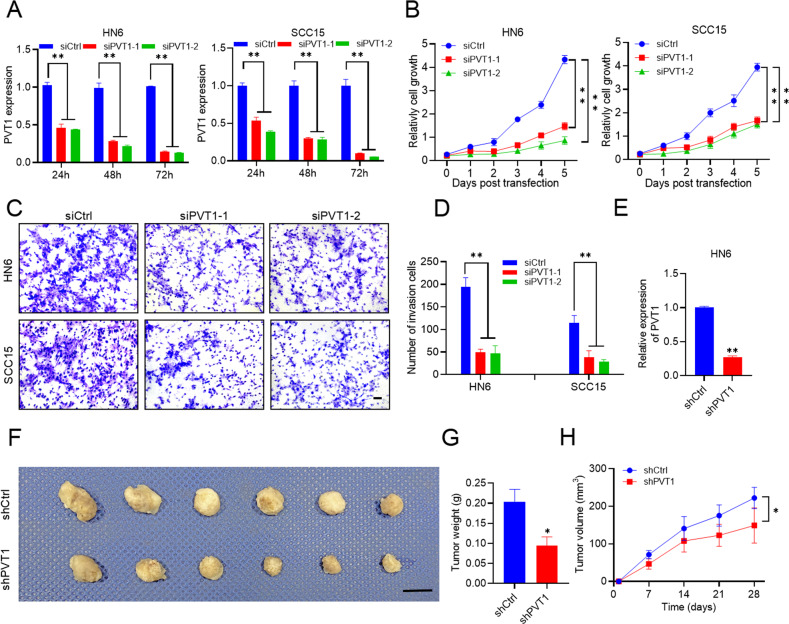
Knockdown of PVT1 inhibits HNSCC proliferation and invasion. **A** qRT-PCR assessment of PVT1 expression in HN6 and SCC15 cells transfected with the siRNA control (siCtrl), siRNA PVT1-1 (siPVT1-1), and siRNA PVT1-2 (siPVT1-2) for 24, 48, and 72 h. Data are shown as the mean ± SD. **p < 0.01 using an unpaired Student’s t-test. **B** The impact of PVT1 knockdown (KD) on HN6 and SCC15 cell proliferation. Data are shown as the mean ± SD. **p < 0.01 using an unpaired Student’s t-test. **C**, **D** The impact of PVT1 KD on HN6 and SCC15 cell invasion after 24 h. Scale bar: 100 μm. Data are shown as the mean ± SD. **p < 0.01 using an unpaired Student’s t-test. **E** qRT-PCR assessment of PVT1 expression in HN6 cells infected with shRNA control (shCtrl) and shRNA PVT1 (shPVT1). Data are shown as the mean ± SD. **p < 0.01 using an unpaired Student’s t-test. **F** Image of the nude mice subcutaneous tumor model established using HN6 cells with PVT1 KD. Scale bar: 1 cm. **G** The weight of PVT1 KD HN6 tumors in nude mice. Data are shown as the mean ± SD (*n* = 6). *p < 0.05 using an unpaired Student’s t-test. **H** The volume of PVT1 KD HN6 tumors in nude mice. Data are shown as the mean ± SD (*n* = 6). *p < 0.05 using an unpaired Student’s t-test.

To explore the function of *PVT1* in HNSCC, two small interfering RNAs, siPVT1-1 and siPVT1-2, were used to specifically knockdown (KD) the expression of PVT1 in HN6 and SCC15 cells (Fig. 2A). PVT1 KD significantly inhibited HN6 and SCC15 proliferation (Fig. 2B). Transwell invasion assay confirmed that PVT1 KD reduced the invasive abilities of HN6 and SCC15 cells (Fig. 2C, D). To investigate whether the PVT1 KD affects tumor development in vivo, we used a lentivirus-based short-hairpin RNA (shPVT1) to knockdown PVT1 in HN6 cells (Fig. 2E). shPVT1-mediated KD of PTV1 significantly reduced the weight and volume of HN6-derived tumors in nude mice compared with that achieved by control shRNA (shCtrl) transfected cells (Fig. 2F–H).

### PVT1 KD suppresses the stemness and metastasis of HNSCC cells

**Fig. 3:**
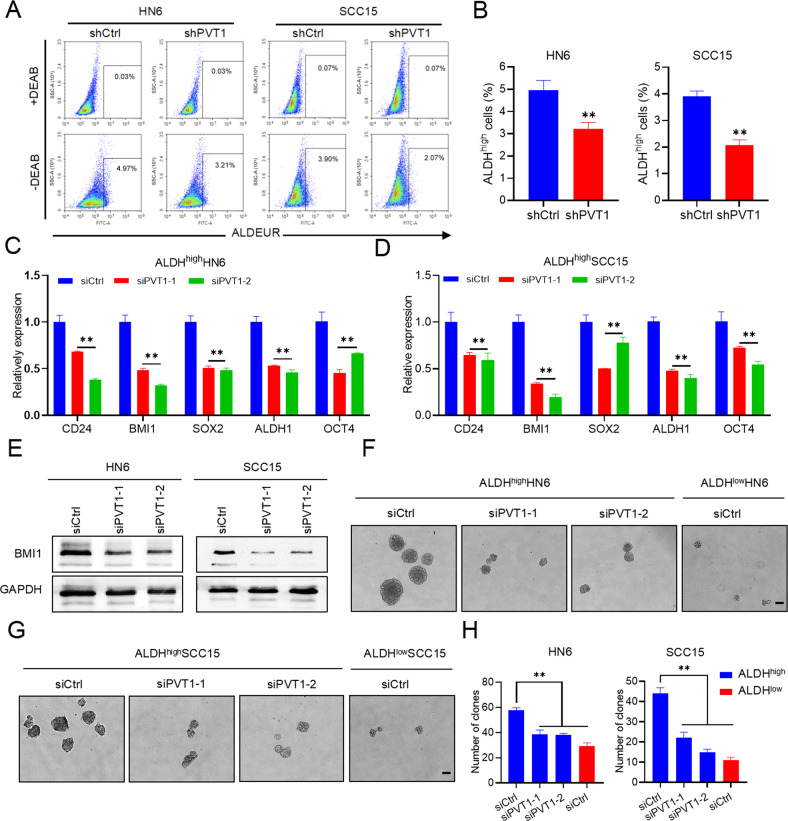
PVT1 KD suppresses cancer stemness. **A**, **B** An ALDEFLUOR assay was conducted in HN6 and SCC15 cells with PVT1 KD, and flow cytometry was used to quantify the percentage of ALDH^high^ cells. Data are shown as the mean ± SD. **p < 0.01 using an unpaired Student’s t-test. **C**, **D** qRT-PCR assessment of stemness-related marker expression in ALDH^high^ HN6 and SCC15 cells with PVT1 KD. Data are shown as the mean ± SD. **p < 0.01 using an unpaired Student’s t-test. **E** Western blot analysis of BMI1 in HN6 and SCC15 cells with PVT1 KD. **F**, **G** Image of tumorspheres formed by ALDH^high^ and ALDH^low^ HNSCC cells transfected with the indicated siRNAs. Scale bar: 100 μm. **H** The numbers of tumorspheres formed by ALDH^high^ and ALDH^low^ HNSCC cells transfected with the indicated siRNAs. Data are shown as the mean ± SD. **p < 0.01 using an unpaired Student’s t-test.

To determine if PVT1 KD could eliminate CSCs, the proportion of ALDH^high^ cells in HN6 and SCC15 cells with PVT1 KD were assessed. Fluorescence-activated cell sorting (FACS) showed that PVT1 KD reduced the number of CSC-like cells among HN6 and SCC15 cells significantly (Fig. 3A, B). The mRNA expression of CSC-related genes, including *CD24*, *BMI1*, *SOX2*, *ALDH1*, and *OCT4*, were reduced in ALDH^high^ HN6 and SCC15 cells with PVT1 KD (Fig. 3C, D). Consistently, the protein expression of these CSCs biomarkers were also reduced after PVT1 KD (Fig. 3E and Supplemental Fig. S1B). CSCs are capable of self-renewal, and spheroid formation is an indicator of self-renewal; therefore, a tumorsphere formation assay was carried out to test the HNSCC self-renewal capabilities. PVT1 KD inhibited the size and number of the ALDH^high^ cell spheres (Fig. 3F–H).

**Fig. 4:**
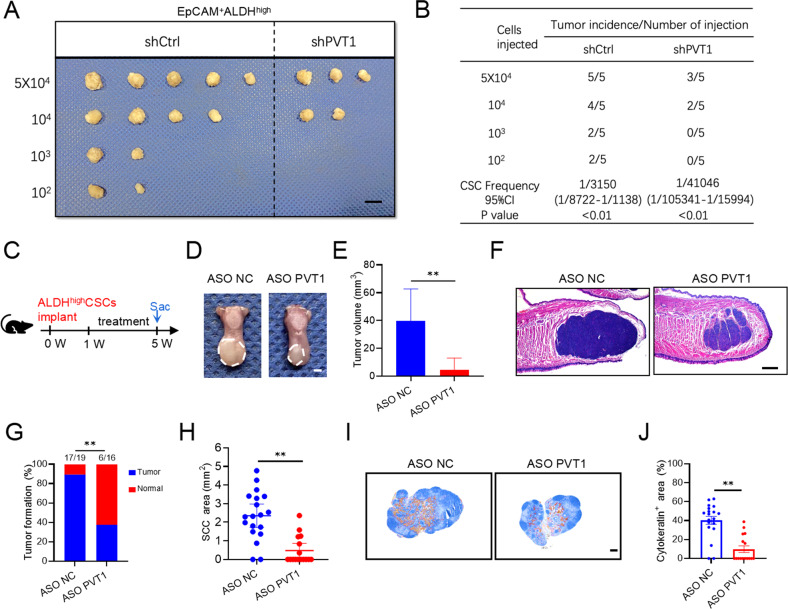
PVT1 KD suppresses the metastasis of HNSCC cells. **A**, **B** Extreme limiting dilution analysis (ELDA) of EpCAM^+^ ALDH^high^ tumor cells transfected with shCtrl and shPVT1 in vivo (*n* = 5). The frequency of allograft formation was displayed for each cell dose injected. ELDA software was used to analyze the data. Scale bar: 1 cm. **C** Schematic diagram of the time points of ASO PVT1 treatment and sacrifice (Sac) of mice injected with ALDH^high^ CSC-like cells from HN6 cells. **D** Representative image of nude mice orthotopic tumors in the tongue from the mice in the different treatment groups. The lesion areas are circled with a white dotted line. Scale bar, 2 mm. **E** Quantification of the tumor volume of nude mice in the different treatment groups. Data are shown as the mean ± SD from at least two independent experiments. **p < 0.01 using an unpaired Student’s t-test. **F** H&E-stained HNSCC from nude mice in the different treatment groups. Scale bar, 500 μm. **G** Quantification of the number of tumors in the nude mice in the different treatment groups. Data are shown as the mean ± SD from at least two independent experiments. **p < 0.01 using a χ^2^ test. **H** Quantification of the area of the tumors in the nude mice in the different treatment groups. Data are shown as the mean ± SD from at least two independent experiments. **p < 0.01 using an unpaired Student’s t-test. **I** A representative image of anti-PCK immunostaining of cervical lymph nodes. Scale bar, 200 μm. **J** Quantification of lymph node metastatic areas of nude mice in the indicated treatment groups. Data are shown as the mean ± SEM from at least two independent experiments. **p < 0.01 using an unpaired Student’s t-test.

Next, CSCs were isolated from a human patient-derived xenograft (PDX) model of HNSCC employing EpCAM^+^ ALDH^high^ markers, as described previously [6]. An in vivo limiting dilution assay showed that PVT1 KD reduced the tumorigenic potential of EpCAM^+^ ALDH^high^ cells from patient case #1-derived PDX (PDX-1) (Fig. 4A, B).

These findings suggested that PVT1 regulates the pro-tumorigenic potential and self-renewal of CSCs, and is associated with lymph node metastasis. A previous study reported that an antisense oligonucleotide (ASO) that could robustly knockdown PVT1 expression [18]. Because subcutaneous tumor animal models are not suitable to assess tumor metastasis, we examined whether PVT1 KD retained the CSCs ability to develop HNSCC and metastasize by a mouse orthotopic HNSCC model [6, 12]. ALDH^high^ CSC-like cells were isolated from HN6 cells and inoculated sublingually into mice tongues. Mice were randomly assigned into two groups and treated with a PVT1 inhibitor (ASO PVT1) or a control ASO (ASO NC) by intratumor injection twice weekly for 4 weeks (Fig. 4C). The resutls showed that the orthotopic tumors derived from the control group were larger than those from the PVT1 KD group (Fig. 4D, E). Histology demonstrated a higher rate of orthotopic tumor formation in the control group than in the PVT1 KD group (Fig. 4F–H). We immunostained cervical lymph nodes using anti-pan-cytokeratin (anti-PCK), which can specifically detect lymph node metastatic tumor cells [19]. Anti-PCK immunostaining showed that PVT1 KD inhibited ALDH^high^ CSC-like cells metastasis to lymph node (Fig. 4I, J).

### PVT1 inhibition eliminates CSCs and stimulates anti-tumor immunity in an immunocompetent microenvironment

**Fig. 5:**
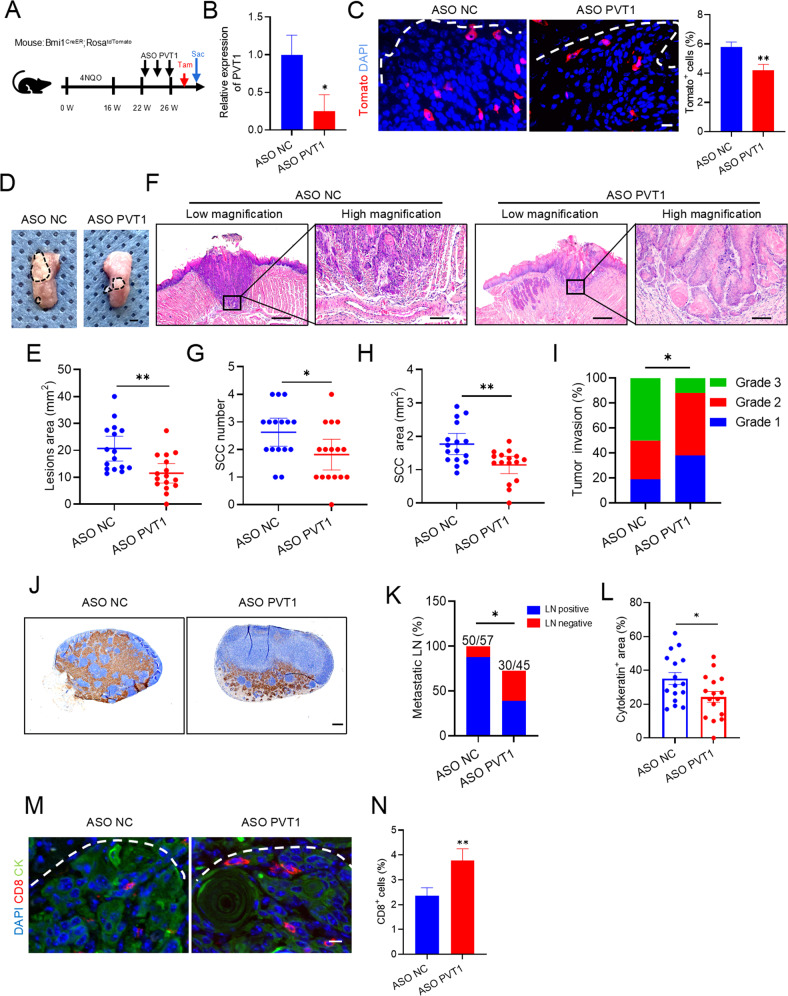
PVT1 inhibition eliminates CSCs and stimulates anti-tumor immunity in an immunocompetent microenvironment. **A** A schematic diagram of the experimental design for treatment and lineage tracing of primary HNSCC in Bmi1^CreER^; Rosa^tdTomato^ mice. For labeling the Bmi1^+^ CSCs, mice were injected with 2.5 mg Tamoxifen (Tam) in a single dose before sacrificing (Sac) mice. **B** qRT-PCR analysis of PVT1 expression in ASO NC and ASO PVT1. Data are shown as the mean ± SD. *p < 0.05 by unpaired Student’s t-test. **C** Representative images of mice with Tomato^+^ Bmi1^+^ CSCs in HNSCC from different treatment groups as indicated. The white dotted line indicates the boundary between the tumor and interstitial tissue. Scale bar, 25 μm. Percentage of Tomato^+^ Bmi1^+^ CSCs in HNSCC from different treatment groups as indicated. Data are shown as the mean ± SD from at least two independent experiments. *n* = 16 **p < 0.01 by unpaired Student’s t-test. **D** Representative image of tumors in tongue from the different treatment groups. The lesion areas were circled with a black dotted line. Scale bar, 2 mm. **E** Quantification of the lesion area of mice with different treatment as indicated. Data are shown as the mean ± SD from at least two independent experiments. *n* = 16. **p < 0.01 by unpaired Student’s t-test. **F** Representative H&E-stained HNSCC of mice with different treatment as indicated. Low-magnification images are shown in the left panels. Scale bar, 500 μm. High-magnification images are shown in the right panels. Scale bar, 100 μm. **G**, **H** Quantification of tumor number and area in mice in the different treatment groups. Data are shown as the mean ± SD from at least two independent experiments. *n* = 16. *p < 0.05 and **p < 0.01 using an unpaired Student’s t-test. **I** Quantification of the grades of HNSCC invasion in mice from the different treatment groups. Results are presented from two independent experiments. *n* = 16. *p < 0.05 using a Cochran-Armitage test. **J** Image of anti-PCK immunostaining of cervical lymph nodes. Scale bar, 200 μm. **K** Proportion of mice with lymph node metastasis from the different treatment groups. The number of metastatic lymph nodes is shown. Results are presented from two independent experiments. *p < 0.05 using a χ^2^ test. **L** Quantification of the metastatic areas in lymph nodes from mice in the indicated treatment groups. Data are shown as the mean ± SEM from at least two independent experiments. *p < 0.05 using an unpaired Student’s t-test. **M** Images of immunofluorescence for CD8^+^ T cells from mice in the different treatment groups. The white dotted line indicates the boundary between interstitial tissue and the tumor. Scale bar, 10 μm. **N** Proportion of immunofluorescence for CD8^+^ T cells from mice in the different treatment groups. Data are shown as the mean ± SD from at least two independent experiments. *n* = 16. **p < 0.01 using an unpaired Student’s t-test.

According to PVT1 function in tumor stemness and metastasis, we assessed PVT1 as a potential HNSCC therapeutic target in an immunocompetent tumor immune microenvironment. In a previous study, our group constructed a 4-Nitroquinoline 1-oxide (4NQO)-induced HNSCC Bmi1^CreER^; Rosa^tdTomato^ mouse model, which permits lineage tracing of CSCs in an unperturbed in vivo environment utilizing induced Cre-mediated recombination induced by tamoxifen [4]. The Bmi1^CreER^; Rosa^tdTomato^ mice received initial 4NQO treatment for 16 weeks, followed by normal drinking water for 6 weeks, to allow HNSCC to develop. And 22 weeks later, they were treated with ASO PVT1 or ASO NC twice weekly for 4 weeks (Fig. 5A). Upon ASO treatment, qRT-PCR analysis showed that PVT1 mRNA expression in the tongue epithelium was significantly reduced in the ASO PVT1 mice compared with that in the ASO NC mice (Fig. 5B). One day before euthanasia, the mice received a single dose of tamoxifen to label the Bmi1^+^ CSCs. Lineage tracing showed that ASO PVT1 significantly reduced the number of Bmi1^+^ CSCs in HNSCC (Fig. 5C). Histological examination demonstrated that PVT1 inhibition inhibited tumor cell numbers, areas, and invasiveness (Fig. 5D–I). Moreover, the metastasis of isolated cervical lymph nodes was compared between the groups. The results showed PVT1 inhibition significantly reduced HNSCC cervical lymph node metastasis in mice (Fig. 5J–L).

The initiation, development, and metastasis of HNSCC are characterized by immune evasion and cancer cell stemness. Targeting the properties of CSCs can be exploited to potentiate immunotherapy [4, 6, 11, 20]. Thus, we hypothesized that PVT1 inhibition might help to stimulate anti-tumor immunity in HNSCC in addition to eliminating CSCs. Immunofluorescence staining confirmed that PVT1 inhibition increased the number of tumor-infiltrating CD8^+^ T cells (Fig. 5M, N).

### Targeting PVT1 to overcome resistance to PD1 blockade therapy and eliminate CSCs

**Fig. 6:**
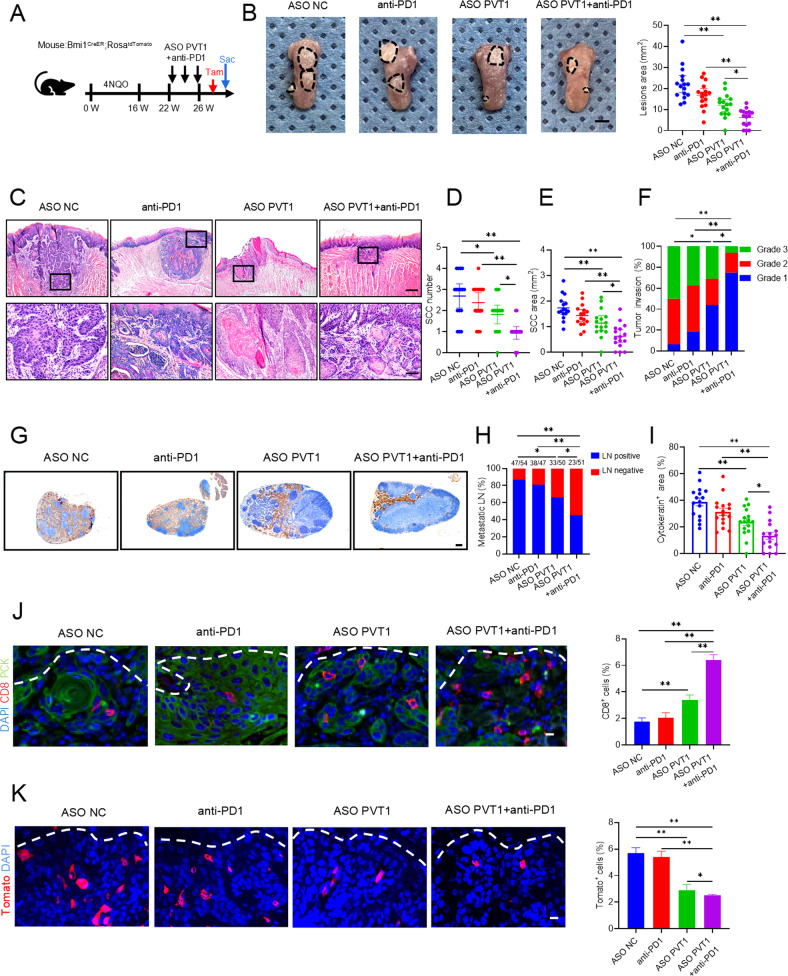
Targeting PTV1 to overcome PD1 blockade resistance and to eliminate CSCs. **A** A schematic diagram of the experimental design for the treatment and lineage tracing of primary HNSCC in Bmi1^CreER^; Rosa^tdTomato^ mice. To label the Bmi1^+^ CSCs, mice were injected with 2.5 mg Tamoxifen (Tam) in a single dose before sacrificing (Sac). **B** Image of tumors in tongues from the different treatment groups. The lesion areas are circled using a black dotted line. Scale bar, 2 mm. Quantification of the HNSCC lesion area of mice with different indicated treatments. Data are shown as the mean ± SD from at least two independent experiments. *n* = 16. *p < 0.05 and **p < 0.01 using one-way ANOVA. **C** Images of H&E-stained HNSCC of mice receiving different indicated treatments. Scale bar, 500 μm. Lower panels show higher magnification images. Scale bar, 100 μm. **D**, **E** Quantification of the tumor number and area of mice in the different treatment groups. Data are shown as the mean ± SD from at least two independent experiments. *n* = 16. *p < 0.05 and **p < 0.01 using one-way ANOVA. **F** Quantification of the grades of HNSCC invasion in mice from the different treatment groups. The results are presented from two independent experiments. *n* = 16. *p < 0.05 and **p < 0.01 using a Cochran–Armitage test. **G** A representative image of anti-PCK immunostaining of cervical lymph nodes. Scale bar, 200 μm. **H** Proportion of mice with lymph node metastasis in the different treatment groups. The number of metastatic lymph nodes is shown. Results are presented from two independent experiments. *p < 0.05 and **p < 0.01 using a χ^2^ test. **I** Quantification of lymph node metastatic areas of mice in the different treatment groups. Data are shown as the mean ± SEM from at least two independent experiments. *p < 0.05 and **p < 0.01 using one-way ANOVA. **J** Immunofluorescent images for CD8^+^ T cells from mice in the different treatment groups. The white dotted line indicates the boundary between interstitial tissue and the tumor. Scale bar, 10 μm. Percentage of immunofluorescence for CD8^+^ T from mice in the different treatment groups. Data are shown as the mean ± SD from at least two independent experiments. *n* = 16. **p < 0.01 using one-way ANOVA. **K** Images of mice with Tomato^+^ Bmi1^+^ CSCs in HNSCC from the different treatment groups. The white dotted line indicates the boundary between interstitial tissue and the tumor. Scale bar, 25 μm. Percentage of Tomato^+^ Bmi1^+^ CSCs in HNSCC from the different treatment groups. Data are shown as the mean ± SD from at least two independent experiments. *n* = 16. *p < 0.05 and **p < 0.01 using one-way ANOVA.

PVT1 inhibition could induce tumor immune response, indicating that in vivo ASO-mediated inhibition of PVT1 might potentiate PD1 blockade. We investigated the combined therapeutic effects of ASO PVT1 plus anti-PD1 (Fig. 6A). Whereas the treatment of ASO PVT1 alone reduced the lesion areas, anti-PD1 did not show the inhibition compared with vehicle control. The addition of anti-PD1 to ASO PVT1 further enhanced the inhibitory effects compared with ASO PVT1 (Fig. 6B). Hematoxylin and eosin (HE) staining showed that the combined treatment reduced the number, area, and invasiveness of HNSCC compared with ASO PVT1 treatment alone (Fig. 6C–F). The anti-PCK immunostaining showed that lymph node metastasis was further inhibited by ASO PVT1 plus anti-PD1 (Fig. 6G–I). ASO PVT1 induced CD8^+^ T cell infiltration, but anti-PD1 did not. By contrast, the combined treatment further increased CD8^+^ T cell infiltration in HNSCC (Fig. 6J). Lineage-tracing revealed that ASO PVT1 plus anti-PD1 reduced more Bmi1^+^ CSCs compared with ASO PVT1 alone (Fig. 6K).

### PVT1 facilitates the cellular DNA damage response and functions as a miRNA sponge for miR-375

**Fig. 7:**
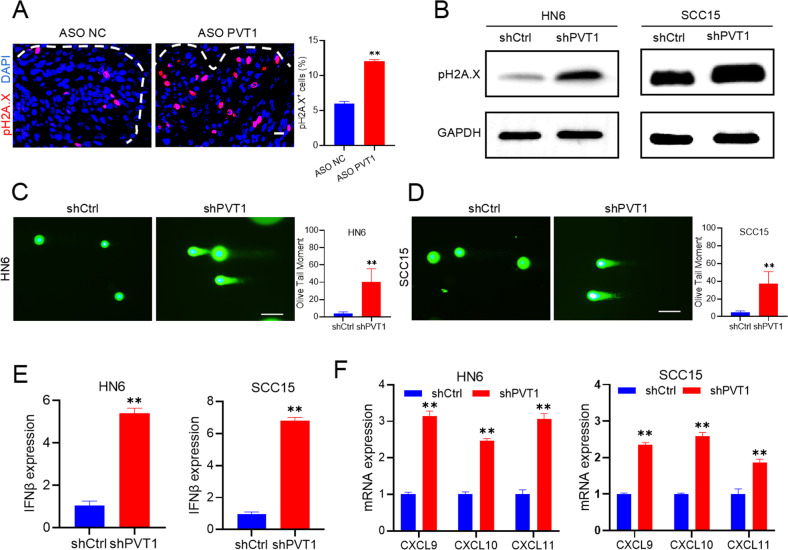
PVT1 facilitates cellular DNA damage response. **A** Immunofluorescent staining and quantification of pH2A.X (red) in 4NQO-induced HNSCC treated with ASO PVT1. DAPI stained the nuclei blue. The white dotted line indicates the boundary between interstitial tissue and the tumor. Scale bar, 25 μm. Data are shown as the mean ± SD. *n* = 16. **p < 0.01 using an unpaired Student’s t test. **B** Western blot of pH2A.X in HN6 and SCC15 cells with PVT1 KD. **C**, **D** Images and quantification of DNA Comet assays in HN6 and SCC15 cells treated with shPVT1 (>10 cells per group). Data are shown as the mean ± SD. Scale bar, 100 μm. **p < 0.01 using an unpaired Student’s t test. **E** qRT-PCR analysis of IFNβ mRNA expression in HN6 and SCC15 cells with PVT1 KD. Data are shown as the mean ± SD. **p < 0.01 using an unpaired Student’s t test. **F** qRT-PCR assessment of the expression levels of CXCL9, CXCL10, and CXCL11 in HN6 and SCC15 cells with PVT1 KD. Data are shown as the mean ± SD. **p < 0.01 using an unpaired Student’s t-test.

A previous study showed that PVT1 participates in DNA damage response and repair regulation [21]. To determine the underlying molecular mechanisms of CD8^+^ T cell recruitment after PVT1 inhibition leading to the promotion of PD1 blockade therapy, we used antiphospho Histone H2A.X (pH2A.X), a DNA damage-specific marker [22]. Immunostaining showed that pH2A.X levels were significantly increased in HNSCC treated with ASO PVT1 (Fig. 7A). Similarly, Western blot showed that PVT1 KD increased pH2A.X levels in HN6 and SCC15, suggesting that inhibition of PVT1 induced DNA damage in HNSCC cells (Fig. 7B). Moreover, in an alkaline comet assay to detect double-stranded DNA (dsDNA) damage, shPVT1 treatment increased the olive tail moment in HN6 and SCC15 cells significantly (Fig. 7C, D). The cGAS-STING (cyclic GMP-AMP synthase-stimulator of interferon genes) signaling axis can be activated by cytosolic dsDNA, which then induces the transcription of IFN and IFN-regulated chemokines [23]. As expected, upon PVT1 KD, the expression of IFNβ significantly increased (Fig. 7E), as did the IFN-regulated chemokines (CXCL9, CXCL10, and CXCL11) (Fig. 7F), which promoted CD8^+^ T lymphocyte recruitment into tumors [24–26]. Moreover, the enzyme-linked immunosorbent assay (ELISA) also confirmed that the protein levels of IFNβ, CXCL9, CXCL10, and CXCL11 from HNSCC cells were induced by PVT1 KD (Supplemental Fig. S3A, B).

**Fig. 8:**
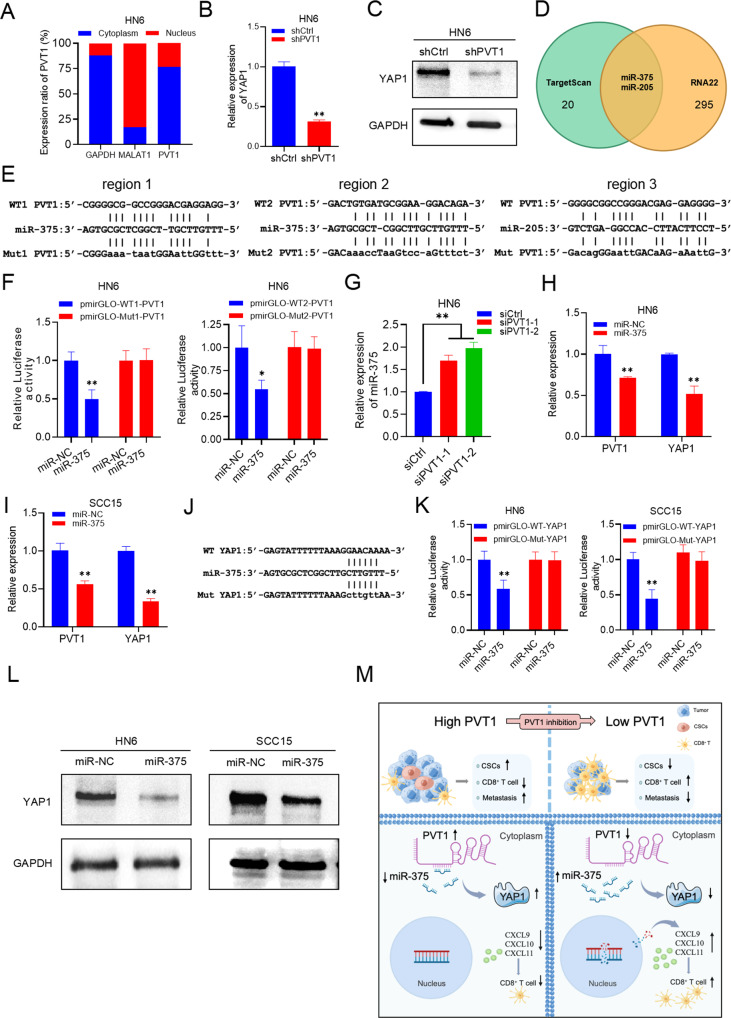
PVT1 functions as a miRNA sponge for miR-375. **A** The subcellular localization of PVT1 by qRT-PCR analysis in HN6 cells. MALAT1 and GAPDH transcripts were used as controls for the nuclear and cytoplasmic fractions. **B** qRT-PCR analysis of YAP1 expression in HN6 cells with PVT1 KD. Data are shown as the mean ± SD. **p < 0.01 using an unpaired Student’s t-test. **C** Western blot analysis of YAP1 in HN6 cells with PVT1 KD. **D** The overlapping miRNAs targeted by PVT1 and YAP1 from two online analysis tools, TargetScan and RNA22, were identified using a Venn diagram. **E** Bioinformatic analysis of miR-375/miR-205 binding to PVT1. **F** The relative luciferase activity in HN6 cells transfected with pmirGLO-WT1-PVT1, pmirGLO-WT2-PVT1, pmirGLO-Mut1-PVT1, pmirGLO-Mut2-PVT1, and miR-375 mimics (miR-375). Data are shown as the mean ± SD. *p < 0.05 and **p < 0.01 using an unpaired Student’s t-test. **G** qRT-PCR assessment of miR-375 expression in HN6 cells transfected with siPVT1-1/2. Data are shown as the mean ± SD. **p < 0.01 using an unpaired Student’s t-test. **H** qRT-PCR assessment of PVT1 and YAP1 expression levels in HN6 cells transfected with miR-375. Data are shown as the mean ± SD. **p < 0.01 using an unpaired Student’s t-test. **I** qRT-PCR assessment of PVT1 and YAP1 expression in SCC15 cells transfected with miR-375. Data are shown as the mean ± SD. **p < 0.01 using an unpaired Student’s t-test. **J** Bioinformatic analysis of miR-375 binding to YAP1. **K** The relative luciferase activity in HN6 and SCC15 cells transfected with pmirGLO-WT-YAP1, pmirGLO-Mut-YAP1 and miR-375. Data are shown as the mean ± SD. **p < 0.01 using an unpaired Student’s t-test. **L** Western blot analysis of YAP1 levels in HN6 and SCC15 cells transfected with miR-375. **M** A diagram shows that the PVT1 inhibition in HNSCC stimulates anti-tumor immunity, prevents metastasis, and depletes CSCs.

To explore the molecular mechanism of PVT1 effect on HNSCC cancer cell stemness, we investigated the subcellular localization of PVT1 using a nuclear and cytoplasmic separation assay. The results showed that PVT1 was predominantly located in the cytoplasm (Fig. 8A and Supplemental Fig. S4A). Given its cytoplasmic localization, PVT1 was likely to sponge miRNAs and release miRNA-targeted mRNA, consequently modulating protein translation. Studies have shown that PVT1 participates in multiple signaling pathways in cancer stemness, including the STAT3, PI3K/AKT, YAP1, and TGFβ pathways [17, 27–29]. Among them, YAP1 expression was one of the most significantly reduced genes in HN6 and SCC15 after PVT1 KD (Fig. 8B, C and Supplemental Fig. S4B, C). Thus, we postulated that *PVT1* could modulate the expression of YAP1 depending, at least in part, on its miRNA sponge function.

Two of the most commonly used software algorithms, RNA22 and Targetscan, were used to predict the binding sites of miRNAs to PVT1 and YAP1. Taking the intersection of the results of the two databases, miR-375 and miR-205 were proposed as candidate miRNAs (Fig. 8D). Next, we constructed luciferase reporter plasmids with part of the PVT1 sequence containing the putative miR-375 and miR-205 binding sites (Fig. 8E). We observed that the luciferase activity of the plasmid containing wild-type (WT) miR-375 binding sites (region 1 or region 2), but not that of the plasmid containing the mutant (Mut) sequences, was suppressed by miR-375 (Fig. 8F and Supplemental Fig. S4D, E). In contrast, there was no effect of miR-205 overexpression on the luciferase activity of the plasmid containing predicted WT binding sequences (region 3) or Mutant miR-205 binding sequences (Supplemental Fig. S4F, G). These results indicated that PVT1 might regulate miR-375, but not miR-205, in HNSCC cells. Next, we narrow down the potential binding sites of miR-375 in PVT1 to a core region. We re-constructed luciferase reporter plasmids containing a series site-directed mutations. For luciferase reporter plasmid containing “region 1”, two putative miR-375-binding sites located at −53/−59 bp and −63/−70 bp of the PVT1 were designated as Mut1a and Mut1b. miR-375 suppressed luciferase activity of the reporter containing the binding sequences of Mut1b, but not the reporter containing the binding sequences of Mut1a (Supplemental Fig. S5A, B). For luciferase reporter plasmid containing “region 2”, two putative miR-375-binding sites located at −1105/−1116 bp and −1117/−1123 bp of the PVT1 were designated as Mut2a and Mut2b. miR-375 suppressed luciferase activity of the reporter containing the binding sequences of Mut2b, but not the reporter containing the binding sequences of Mut2a (Supplemental Fig. S5C, D). These results indicated that −53/−62 bp and −1106/−1116 bp of the PVT1 were the core binding sites of miR-375. Moreover, miR-375 levels were increased in cells after PVT1 KD (Fig. 8G and Supplemental Fig. S4H). Transfection of miR-375 mimics into HN6 or SCC15 cells resulted in significant downregulation of PVT1 and YAP1 (Fig. 8H, I and Supplemental Fig. S4I). *YAP1* is a known oncogene targeted by miR-375 in lung cancer [30], gastric cancer [31], and prostate cancer [32]. The luciferase reporter plasmids comprising the WT or mutant-type miR-375 target site from the 3′UTR of YAP1 were constructed [33] (Fig. 8J). The luciferase activity of the WT reporter plasmids, but not that of the mutant reporter plasmids, was suppressed after miR-375 overexpression (Fig. 8K). Consistently, overexpression of miR-375 also reduced the protein level of YAP1 in HNSCC cells (Fig. 8L). Thus, our results demonstrated that PVT1 positively regulated YAP1 expression via its ceRNA activity toward miR-375. We also performed rescue assays to evaluate whether miR-375 is necessary for PVT1-regulated cancer stemness and immune evasion. The results showed that miR-375 overexpression reversed the PVT1 promotion effects on HN6 and SCC15 proliferation and invasion (Supplemental Fig. S6A–C). Whereas PVT1 increased the size and number of the ALDH^high^ cell spheres, ectopic expression of miR-375 reversed this effect (Supplemental Fig. S6D). qRT-PCR and ELISA assays demonstrated that miR-375 overexpression counteracted the effect of PVT1 on the expression of IFNβ, CXCL9, CXCL10, and CXCL11 in HNSCC cells (Supplemental Fig. S6E, F).

## Discussion

Growing evidence demonstrates that cancer stemness and immune evasion play a critical role in tumor development, progression, and metastasis. LncRNAs have been shown to be important regulators of stemness maintenance and the immune response through various functions, such as affecting chromatin epigenetic modification, protein complex composition, and protein translation or degradation [34–37]. However, their mechanisms have not been clarified. Herein, the stemness and immune evasion of HNSCC were characterized molecularly. In this study, PVT1 was observed to be highly expressed in HNSCC and closely correlated with metastasis. PVT1 KD inhibited HNSCC cell proliferation, invasion, reduced stemness, and delayed the in vivo and in vitro growth of tumors. Mechanistically, our results revealed that PVT1 inhibition stimulates the DNA damage response, which induces CD8^+^ T cell-recruiting chemokines, while preventing CSCs and metastasis via regulating the miR-375/YAP1 axis (Fig. 8M).

CSCs are thought to be the primary agents responsible for tumor development, metastasis, recurrence, and heterogeneity [38]. LncRNAs have been reported to play widespread roles in gene regulation and self-renewal of cancer cells. Notably, recent studies showed that lncTCF7-mediated Wnt signaling primes liver CSC self-renewal and tumor propagation [34]. These studies show that lncRNAs are indispensable regulatory factors for the maintenance of stemness. In our study, we found that there was significant difference in the expression of PVT1 between ALDH^high^ and ALDH^low^ cells. We showed that PVT1 KD reduced the expression of CSCs markers and the size of tumorspheres, implying that *PVT1* might act as an oncogene in tumors.

In addition to actively invading the human body, tumor cells have also evolved multiple defense strategies to avoid being eradicated by the immune system. Cancer progression is induced by the immune escape of cancer cells from CD8^+^ T cells [39]. LncRNAs have been shown to exert vital functions in activating immune cells. LncRNA *KCNQ1OT1* might affect tumor prognosis through CD8^+^ T cell infiltration in patients with colon adenocarcinoma [40]. An essential mechanism for limiting the growth and spread of tumors is immunosurveillance. Immune checkpoint inhibitors have drastically enhanced cancer treatment. A few PD-1 antibodies have been approved by the FDA to treat recurrent and metastatic HNSCC [41]. Unfortunately, this treatment frequently fails to produce long-lasting results. To obtain a long-lasting cure and relapse-free survival, it is necessary to develop innovative strategies. Cancer-associated genes can be targeted using ASOs [42, 43]. Moreover, cancer therapy could be achieved using combination therapy comprising currently existing anti-cancer drugs and ASOs. In our research, we constructed a spontaneous tumor model. In that model, ASO PVT1 reduced the growth and metastasis of HNSCC, and these effects were enhanced using the combination therapy. Our results indicated that the combination therapy comprising anti-PD1 and ASO PVT1 effectively eliminated CSCs to inhibit tumor growth and metastasis, and activated the tumor immune response to increase the recruitment of CD8^+^ T cells into tumor tissues. These results suggested the importance of lncRNAs in activating intratumoral immunity.

DNA damage is thought to be a weakness of cancer cells because it can induce immune responses that target and eradicate cancer cells [44]. Recent studies have reported that DNA damage-mediated cGAS-STING activation promotes IRF3 and NF-kB transcriptional activation [45, 46]. Therefore, the interplay among the DNA damage response, cGAS-STING pathway activation, and anti-tumoral immunity is critical to reveal novel targets for future cancer immunotherapies [47]. Our study assessed the extent of DNA damage through pH2A.X and comet assays to explore the role of PVT1 in DNA damage. We determined that PVT1 was associated with DNA damage in HNSCC. Our data showed PVT1 KD triggered DNA damage accumulation; mediated the expression and secretion of proinflammatory cytokines and proinflammatory chemokines (CXCL9, CXCL10, CXCL11); and activated CD8^+^ T cell recruitment, which identified PVT1 as a potential therapeutic target.

According to the ceRNA hypothesis, the shared miRNAs serve as a channel for communication and coregulatory activity between mRNAs, transcribed pseudogenes, and lncRNAs [48]. Herein, most PVT1 was located in the cytoplasm of HNSCC cells. Bioinformatic analysis and luciferase assays revealed that miR-375 is a bridge connecting YAP1 and PVT1. In addition, PVT1 KD decreased YAP1 expression, while the expression of miR-375 exhibited the opposite trend. These data showed PVT1 regulated the miR-375/YAP1 axis. According to our findings, PVT1 inhibited HNSCC stemness, which was supported by the percentage of the EpCAM^+^/ALDH^+^ subpopulation, the expression of stemness markers, and the capacity for sphere formation. The majority of research supports the classification of miR-375 and YAP1 as important tumor suppressors [49–51]. We concluded that PVT1 could prevent stemness and metastasis in HNSCC by regulating the miR-375/YAP1 axis. Therefore, further research is required to explore PVT1 as a bona fide target in cancer therapy based on lncRNAs.

In summary, our findings are important to develop new combination treatments for HNSCC that involve targeting PVT1 to eliminate CSCs, prevent metastasis, and activate the intrinsic immune responses of tumor cells.

## Materials and methods

### Patients and clinical samples

To generate the HNSCC PDX, the human HNSCC tissues were obtained without patient information from the Peking University School and Hospital of Stomatology. The tumor tissues were cut into small pieces, followed by implantation into the flanks of NOD-SCID mice (6 weeks old), according to a previously described method [8]. HNSCC specimens from 60 patients were obtained from the Peking University School and Hospital of Stomatology from September 2012 to October 2016. The inclusion criteria were as follows: 1) the tumor was in the tongue; 2) there was no distant metastasis; 3) removal of the primary carcinoma and neck dissection without preoperative radiotherapy or chemotherapy; and 4) patients who underwent postoperative follow-up for at least five years. Without conducting a pathological study, the clinical TNM staging approach was used to classify the tumor size and clinical stage for the 40 HNSCC samples among the 60 samples: 1) tumor size limited in T2 and T3; 2) clinically negative cervical lymph node (cN0); and 3) no distant metastasis (M0). Based on the histopathologic evaluation of the lymph nodes, these 40 patients were split into lymph node-negative and positive groups. These experiments were approved by the Institutional Review Board of the Peking University School and Hospital of Stomatology and all samples were obtained from patients who signed informed consent forms approving the use of their tissues for research purposes after surgery (Approval number: PKUSSIRB-2012010). The tissues were snap-frozen and placed at −80 °C until analysis.

### 4NQO mouse model of HNSCC, treatment and histology

The Bmi1^CreER^; Rosa^tdTomato^ mice received drinking water with 4NQO for 16 weeks to allow HNSCC to develop, followed by normal drinking water for 6 weeks, to form a spontaneous model of HNSCC. The mice were randomly divided into groups at 22 weeks. Before sacrificing the mice, they were given tamoxifen to label Bmi1^+^ CSCs. For the treatment assay, Bmi1^CreER^; Rosa^tdTomato^ mice were divided randomly into the indicated groups, and injected with the indicated ASO PVT1 (10 nM, Integrated Biotech Solutions Co., Ltd, Shanghai, China) and ASO NC over the whole mouse tongue by twice-weekly subcutaneous injection for 4 weeks. For combination therapy, mice were intraperitoneally injected with anti-PD1 (BioXcell, Lebanon, NH, USA, 200 μg/mouse twice per week). After mice were sacrificed, the cervical lymph nodes and tongues were removed, and the lesion surface areas were calculated. For histological investigation and immunostaining, longitudinally cut tongues (dorsal/ventral) and intact lymph nodes were fixed overnight in 4% paraformaldehyde and paraffin-embedded. 10 sections of 5 mm thick tissue blocks were cut, and they were then stained with hematoxylin and eosin (HE). The SCC number was counted and regions were measured [8]. The following criteria were used to grade the invasiveness of the HNSCC: showing signs of normal or epithelial dysplasia appearance (grade 1); distinct invasion, unclearness of the basement membrane, drop and diffuse infiltration into the superficial portion of the muscle layer (grade 2); loss of the basement membrane; extensive invasion into deep muscle layer (grade 3). To examine cervical lymph node metastasis of HNSCC, the sections of cervical lymph nodes were immunostained with anti-PCK antibodies.

### CSCs isolation and tumorsphere formation assay

An ALDEFLUOR assay kit (Stemcell Technologies, Vancouver, Canada) was used to sort ALDH^high^ CSCs from HN6 and SCC15 cells following the manufacturer’s protocol. Flow cytometry was used to detect green fluorescence-positive cells among live cells, in comparison with the fluorescence intensity of the diethylaminobenzaldehyde (DEAB) treated sample. These detected cells with high ALDH activity (ALDH^high^ cells) were used for subsequent experiments. Human HNSCC xenografted tumors were chopped into small pieces and digested into single-cell suspensions using a human tumor cell dissociation kit (Miltenyi Biotec, Bergisch Gladbach, Germany) to isolate CSCs from human HNSCC PDX. An EpCAM-PE (Miltenyi Biotec) was used to isolate EpCAM^+^ tumor cells, and an ALDEFLUOR assay kit was used to sort the ALDH^high^ and ALDH^low^ subpopulations from the EpCAM^+^ tumor cells by flow cytometry. For the tumorsphere formation assay, ALDH^high^ and ALDH^low^ cells were added to ultralow attachment plates and cultured in DMEM/F12 (Thermo Fisher Scientific, Waltham, MA, USA) without serum but containing 1% B27 supplement (Thermo Fisher Scientific), 1% N2 supplement (Thermo Fisher Scientific), human recombinant epidermal growth factor (EGF, 20 ng/mL, R&D Systems, Minneapolis, MN, USA), and human recombinant basic fibroblast growth factor (bFGF, 10 ng/mL, R&D Systems). After 10 days, spheres with a diameter exceeding 70 μm were counted under a microscope.

### In vivo tumor growth in mice

Female Nude/SCID mice (6–8 weeks old) were obtained from SiPeiFu Biotechnology Co., Ltd (Beijing, China). All mouse studies were performed strictly according to the animal protocol #LA2021020 approved by Peking University Biomedical Ethics Committee. HN6 cells (with or without PVT1 KD) were injected subcutaneously into the backs of the nude mice, after mixing with an equal volume of Matrigel. The Nude/SCID mice were monitored; and the weight and volume of the xenograft tumors were measured using sliding calipers. At 4 weeks after injection, the mice were sacrificed, and the tumors were excised and weighed. For the in vivo limiting dilution assay, EpCAM^+^ ALDH^high^ CSCs were subjected to infection for 24 h with the lentiviruses expressing shPVT1 or shCtrl. Following rapid puromycin selection, varying numbers of cells were mixed with an equal volume with Matrigel and injected subcutaneously into the backs of nude mice for 4 weeks. The Extreme Limiting Dilution Analysis software (http://bioinf.wehi.edu.au/software/elda/) was used to analyze the data. Tumor volume were computed using the formula (L × W^2^)/2 (where L is the longer diameter and W is the shorter diameter). For orthotopic tumor formation, HN6-derived ALDH^high^ CSC-like cells were inoculated sublingually into the tongues of Nude/SCID mice and grew for 1 week. For treatment, tumor-bearing mice were divided randomly into the indicated groups, and injected with the indicated ASO PVT1 (10 nM, Integrated Biotech Solutions Co., Ltd) and ASO NC over the whole mouse tongue by twice-weekly subcutaneous injection for 4 weeks. After mice were sacrificed, the cervical lymph nodes and tongues were removed.

### Immunostaining

4% paraformaldehyde was used to fix mouse HNSCC tongues and cervical lymph nodes for 24 h, followed by equilibration in 30% sucrose/phosphate-buffered saline (PBS), and embedding in optimal cutting temperature compound (Sakura Finetek, Tokyo, Japan). Coronal 5 μm sections were cut on a freezing microtome (Leica, Wetzlar, Germany). Thereafter, sections were stained with 4′,6-diamidino-2-phenylindole (DAPI) solution (Solarbio, Beijing, China). These tissues were embedded in paraffin and cut into 5 µm sections. Sections were deparaffinized with xylene and rehydrated through an ethanol series and distilled water. For immunohistochemistry, antigens were repaired using high temperature and pressure in citrate buffer (pH = 6). Sections were then incubated with the following primary antibodies at 4 °C overnight: anti-PCK (1:200, Santa Cruz, CA, USA, Cat#sc-8018). Next, horseradish peroxidase-labeled polymer was incubated with the sections for 60 min. The signals were detected using 3,3′-Diaminobenzidine as a chromogen (Zhongshan Golden Bridge, Beijing, China), followed by counterstaining using hematoxylin. For immunohistofluorescence, after antigen repair, the sections were incubated at 4 °C overnight with the following primary antibodies: CD8α (1:1000, Cell Signaling Technology, Danvers, MA, USA, Cat#98941), anti-PCK (1:200, Abcam, Cambridge, MA, USA, Cat#ab9377), antiphospho Histone H2A.X (Ser139) (1:200, Cell Signaling Technology, Cat#9718). Thereafter, the sections were visualized using Cy2 and Cy3-conjugated secondary antibodies (Jackson ImmunoResearch Laboratories, West Grove, PA, USA).

### Cell culture, PVT1 KD and overexpression

The American Type Culture Collection (ATCC, Manassas, VA, USA) provided the human HNSCC cell lines SCC15. HN6 was obtained from the Central Laboratory of Peking University School and Hospital of Stomatology. Dulbecco’s modified Eagle’s medium (DMEM) with 10% fetal bovine serum (FBS) and 1% antibiotics was used to culture the cells at 37 °C, 5% CO_2_ in a humidified incubator. Cells were transfected with miRNA mimics (Integrated Biotech Solutions Co., Ltd) or siRNA (Tsingke Biotechnology, Beijing, China) using the Lipo8000^TM^ Transfection Reagent (Beyotime, Shanghai, China) following the manufacturer’s instructions. The lentiviruses used for PVT1 KD or overexpression were from Integrated Biotech Solutions Co., Ltd (Shanghai, China). Briefly, the scramble control or the specific PVT1 KD (shPVT1) and overexpression (pQLL-PVT1) lentiviral plasmids were cotransfected into HEK293T cells with two helper plasmids psPAX2 (Addgene, Watertown, MA, USA Cat#12260) and pMD2.G (Addgene, Cat#12259). Viral supernatants were harvested for cell infection 72 h after transfection. Cells were infected with lentiviruses in the presence of polybrene (Sigma-Aldrich, Shanghai, China, Cat#H9268), selected with puromycin (Sigma-Aldrich, Cat#P9620) and expanded before being used for subsequent assays.

### Western blot

Cells were lysed in Radioimmunoprecipitation assay buffer (Solarbio) containing phenylmethylsulphonyl fluoride protease inhibitor. Protein samples were subjected to 10% SDS polyacrylamide gel electrophoresis. The separated proteins were subsequently transferred to a polyvinylidene fluoride membrane. The membrane was blocked using 10% non-fat milk for 1 h at room temperature and then incubated with primary antibodies overnight at 4 °C. Primary antibodies recognizing the following proteins were used: CD24 (1:1000, Proteintech, Wuhan, China, Cat#18330-1-AP), SOX2 (1:1000, Cell Signaling Technology, Cat#14962), ALDH1 (1:1000, Cell Signaling Technology, Cat#54135), OCT4 (1:1000, Wanlei, Shenyang, China, WL02020), BMI1 (1:1000, Cell Signaling Technology, Cat#6964), YAP1 (1:1000, Cell Signaling Technology, Cat#14074), GAPDH (encoding glyceraldehyde-3-phosphate dehydrogenase) (1:20000, Proteintech, Cat#60004-1). anti-phospho Histone H2A.X (Ser139) (1:1000, Cell Signaling Technology, Cat#9718). Thereafter, the membranes were incubated with secondary antibodies corresponding to the primary antibodies for 1 h at room temperature. Finally, the immunoreactive protein bands were visualized using ECL (NCM Biotech, Suzhou, China) Western blot detection reagent.

### qRT-PCR

Total RNA was isolated using the Trizol reagent (Invitrogen, Waltham, MAS, USA) following the manufacturer’s protocol. RNA (1–2 μg) was reverse transcribed to cDNA using random primers (Takara, Beijing, China). The mRNA levels were quantified using quantitative real-time PCR with the cDNA as the template employing the SYBR Green supermix (Roche, Basel, switzerland). The internal control was GAPDH. Table S1 (Supporting Information) lists the primers used for qRT-PCR.

### Nuclear and cytoplasmic fraction isolation

Nuclear and cytoplasmic RNAs were isolated using a Nuclear and Cytoplasmic Protein Extraction Kit (Beyotime) following the manufacturer’s protocol. Briefly, cell fraction buffer was used to lyse the cells, followed by slow speed centrifugation to separate the nuclear fraction from the cytoplasmic fraction. RNAs from the two fractions were isolated separately using Trizol. MALAT1 and GAPDH transtripts were used as controls for the nuclear and cytoplasmic fractions, respectively.

### Cell proliferation assay and transwell invasion assay

Cells were grown in 96-well plates and transfected with siRNA (siPVT1-1/2). Subsequently, a Cell Counting Kit-8 (CCK-8, Dojindo, Shanghai, China) was used to detect cell proliferation at 0, 24, 48, 72, 96 and 120 h following the manufacturer’s protocol. For Transwell invasion assays, cells were inoculated into Transwell plates containing Matrigel. DMEM with 20% FBS was placed in the lower chamber. Serum-free DMEM was used to resuspend the cells, which were added to the upper chamber. After 24 h, 70% methanol was used to fix the membranes, which were then stained using 0.1% crystal violet for 10 min. After thorough washing with PBS, the membranes were imaged. The average number of invasive cells was determined by counting in three randomly chosen fields under a microscope.

### Luciferase reporter assay

The potential miRNAs binding to PVT1 were predicted using RNA22 (https://cm.jefferson.edu/rna22/Interactive/). The potential miRNAs binding to YAP1 were predicted using Targetscan (https://www.targetscan.org/vert72/). The specific regions of the PVT1 transcript which contained the predicted targets of miR-375/205, were amplified from the cDNA of HN6 cells by PCR and cloned into the pmirGLO Dual-Luciferase Vectors to construct dual-luciferase (firefly and renilla luciferase) reported plasmids. HNSCC cells were grown in a 48-well plate were co-transfected with dual-luciferase reporters (pmirGLO-WT1-PVT1, pmirGLO-WT2-PVT1, pmirGLO-WT-PVT1, pmirGLO-Mut1-PVT1, pmirGLO-Mut2-PVT1, pmirGLO-Mut-PVT1, pmirGLO-WT-YAP1, pmirGLO-Mut-YAP1, pmirGLO-Mut1a-PVT1, pmirGLO-Mut1b-PVT1, pmirGLO-Mut2a-PVT1, pmirGLO-Mut2b-PVT1) and miRNA mimics by Lipo8000^TM^ Transfection Reagent. Subsequently, a Dual-Glo Luciferase Assay System Kit (Beyotime) was used to detect dual-luciferase activity following the manufacturer’s protocol. Renilla luciferase activity was used to normalize the firefly luciferase activity.

### Comet assay

Using a SCGE test Kit (Enzo Life Sciences, Farmingdale, NY, USA), single-cell gel electrophoresis comet experiments were carried out. Following treatment, 75 μl aliquots of the cells were placed onto preheated slides at a volume ratio of 1:50 with low melting point agarose. The slides were incubated in pre-chilled lysis solution for 60 min, and then in prechilled alkaline solution for 30 min. The slides were then subjected to electrophoresis at 25 V in TBE buffer for 20 min. Comets were stained using CYGREEN dye for 30 min and then imaged. CASP Version 1.2.2 analytic tool was used to examine at least 50 distinct cells per sample in duplicate (CASPlab, Wroclaw, Poland).

### ELISA assay

HN6 and SCC15 cells with different treatment were cultured for 48 h. The supernatants were collected and the protein levels of IFNβ, CXCL9, CXCL10 and CXCL11 were detected by ELISA kit (Thermo Fisher Scientific, Cat#414101, R&D Systems, Cat#DCX900, Cat#DIP100, Cat#DCX110) according to the manufacturer’s instructions.

### RNA-seq and analysis

The Trizol reagent was used to isolate total RNAs. An Agilent Bioanalyzer 2100 (Agilent technologies, Santa Clara, CA, USA) was used to assess RNA quality. An RNAClean XP Kit (Beckman Coulter, Brea, CA,USA) and RNase-Free DNase Set (QIAGEN, Hilden, Germany) were used to purify the total RNA. An Illumina TruSeq® RNA sample preparation Kit (Illumina, San Diego, CA, USA) was used to prepare the sequencing libraries, and an Illumina HiSeq 2500 machine was used to pair-end sequence the libraries. GraphPad Prism 9.0 (GraphPad software, Inc., La Jolla, CA, USA) was used to construct a heatmap. We deposited the raw sequencing data at GEO under the accession number GSE210387.

### Statistical analyses

The figure legends show the statistical parameters of the analyses. In vitro experiments were carried out at least three times, and in vivo experiments were carried out at least twice. GraphPad Prism 9.0 for windows (GraphPad software, Inc.) was used to carry out the statistical analyses. Comparisons between the data from two groups were performed using Student’s t-test followed by Tukey’s HSD post hoc tests to minimize type I errors. The survival rates were calculated using the Kaplan–Meier method and analyzed using the log-rank test. For comparison of invasiveness in HNSCC, the differences were evaluated using the Cochran–Armitage test. Among the different treatment groups, the proportion of mice with lymph node metastasis was assessed using a χ^2^ test. One-way analysis of variance (ANOVA) was used to compare the differences in HNSCC lesion size, number, and area in the control and treated mice. In the ANOVA analyses, the Shapiro–Wilk test was used to validate that the data were normally distributed and that all the data satisfied the assumption of no significant outliers. *p* < 0.05 was considered significant.

## Supplementary information

PVT1 Supplemental Figures and Table

PVT1 Supplemental file_Uncropped WB

Reproducibility Checklist

## Data Availability

The data that support the findings of this study are available from the corresponding author LF Jia, upon reasonable request.
