# Supplementary material for: PVT1 inhibition stimulates anti-tumor immunity, prevents metastasis, and depletes cancer stem cells in squamous cell carcinoma
Source: Cell Death Dis. 2023 Mar 9;14(3):187. doi: 10.1038/s41419-023-05710-6 (PMC9998619; doi:10.1038/s41419-023-05710-6)
Supplement: Supplementary file 1 — PVT1 Supplemental Figures and Table [file 41419_2023_5710_MOESM1_ESM.docx]

**PVT1 Inhibition Stimulates Anti-tumor Immunity,** **Prevents Metastasis, and Depletes Cancer Stem Cells in Squamous Cell Carcinoma**

Zhen Qin^1#^, Wenbo Zhang^1#^, Shuo Liu^1^, Yujia Wang^1^, Xin Peng^1^*, Lingfei Jia^1, 2, 3^*

**Affiliation:**

^1^Department of Oral and Maxillofacial Surgery, Peking University School and Hospital of Stomatology, Beijing 100081, China.

^2^Department of Central Laboratory, Peking University School and Hospital of Stomatology, Beijing 100081, China.

^3^National Center for Stomatology & National Clinical Research Center for Oral Diseases & National Engineering Laboratory for Digital and Material Technology of Stomatology, Beijing 100081, China.

^#^These authors contributed equally to this work.

***Corresponding author:**

Lingfei Jia, PhD.

22 Zhongguancun South Avenue, Beijing 100081, China.

E-mail: jialingfei1984@sina.com

Tel: (8610) 82195109

Fax: (8610) 82193402

Xin Peng, MD, DDS.

22 Zhongguancun South Avenue, Beijing 100081, China.

E-mail: pxpengxin@263.net

Tel: (8610) 82195210

Fax: (8610) 62173402

**Supplemental Figures**

**Supplemental Table**


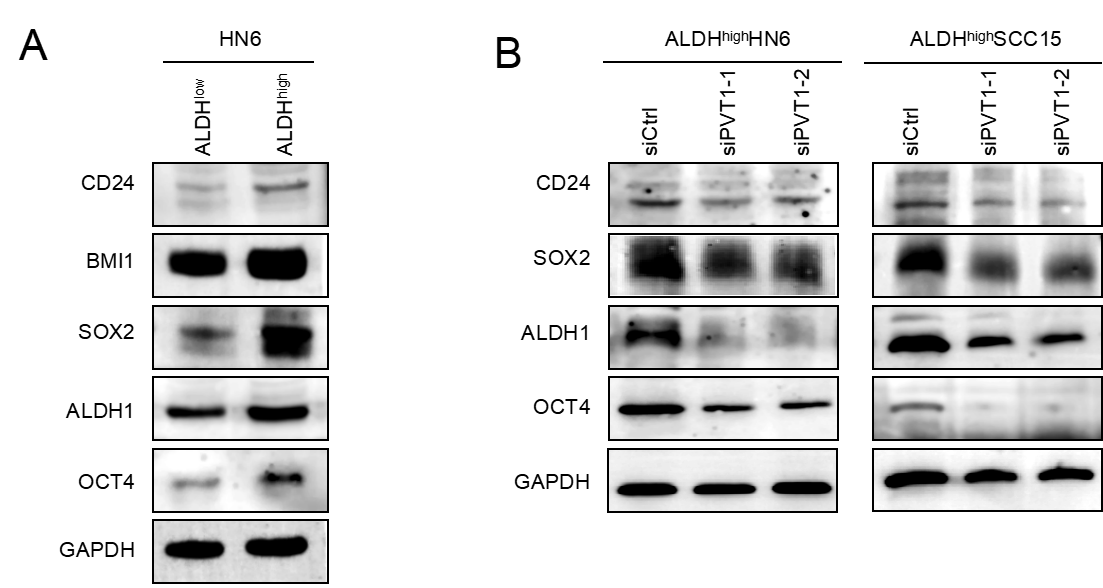


**Supplemental Fig. S1 The protein levels of HNSCC CSC-characteristic genes were increased in the ALDH^high^ CSC-like cells. A** Western blot analysis of stemness-related genes in ALDH^high^ CSCs and ALDH^low^ non-CSCs. **B** Western blot of stemness-related genes in ALDH^high^ HN6 and SCC15 cells with PVT1 KD.

**
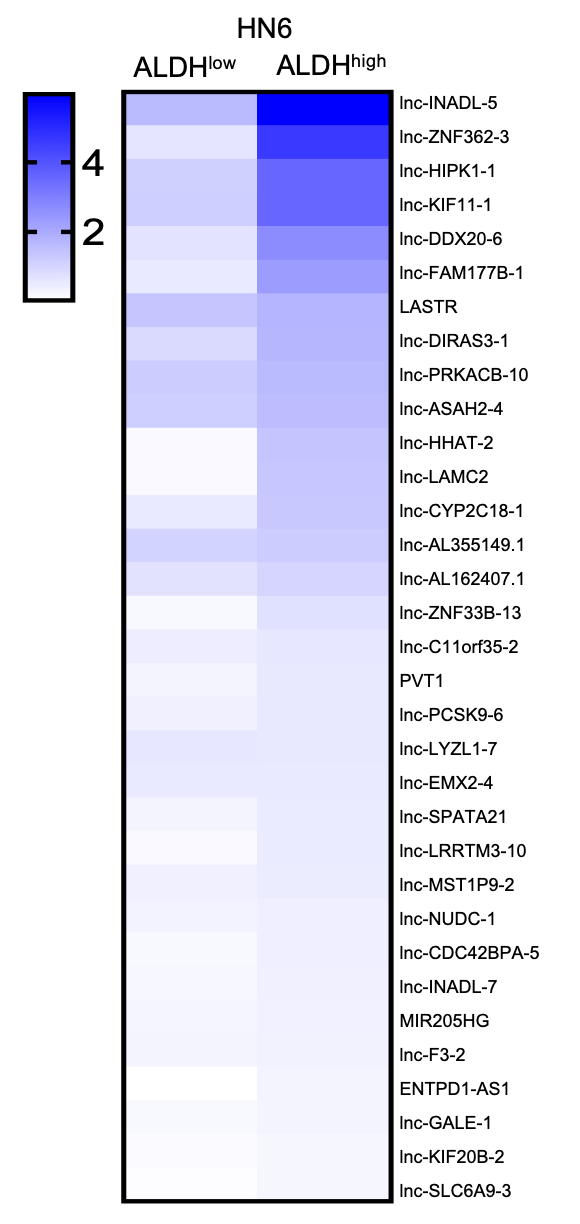
**

**Supplemental Fig. S2** A cluster heatmap (fold change > 3.0, p < 0.01) showing the 33 most upregulated lncRNAs in ALDH^high^ CSCs compared with ALDH^low^ non-CSCs.


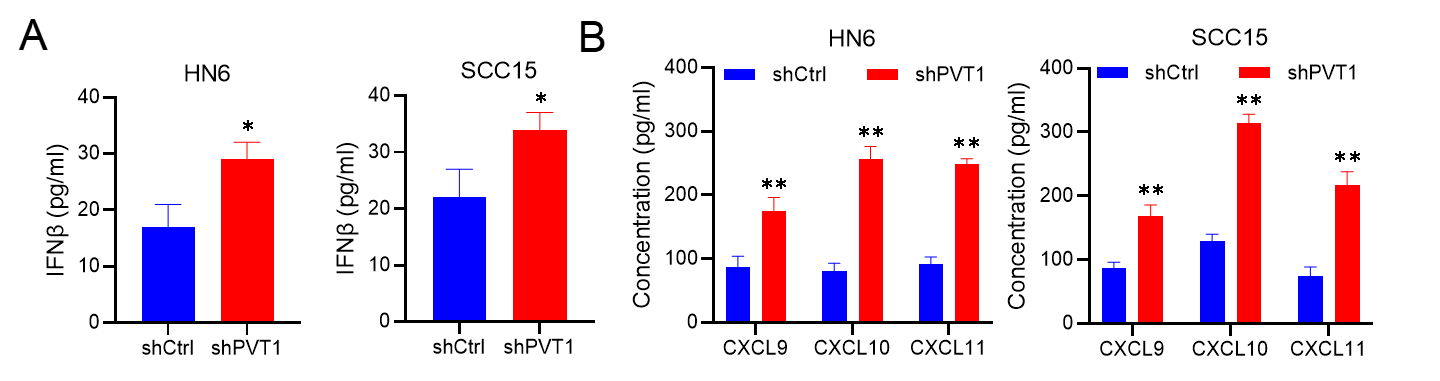


**Supplemental Fig. S3 PVT1 KD increased the levels of IFNβ, CXCL9, CXCL10, and CXCL11 in HN6 and SCC15 cells. A** ELISA analysis of the protein levels of IFNβ secreted by HN6 and SCC15 cells treated with PVT1 KD. Means ± SD are shown. *p < 0.05 by unpaired Student’s t-test. **B** ELISA analysis of the protein levels of CXCL9, CXCL10, and CXCL11 secreted by HN6 and SCC15 cells with PVT1 KD. Means ± SD are shown. **p < 0.01 by unpaired Student’s t-test.


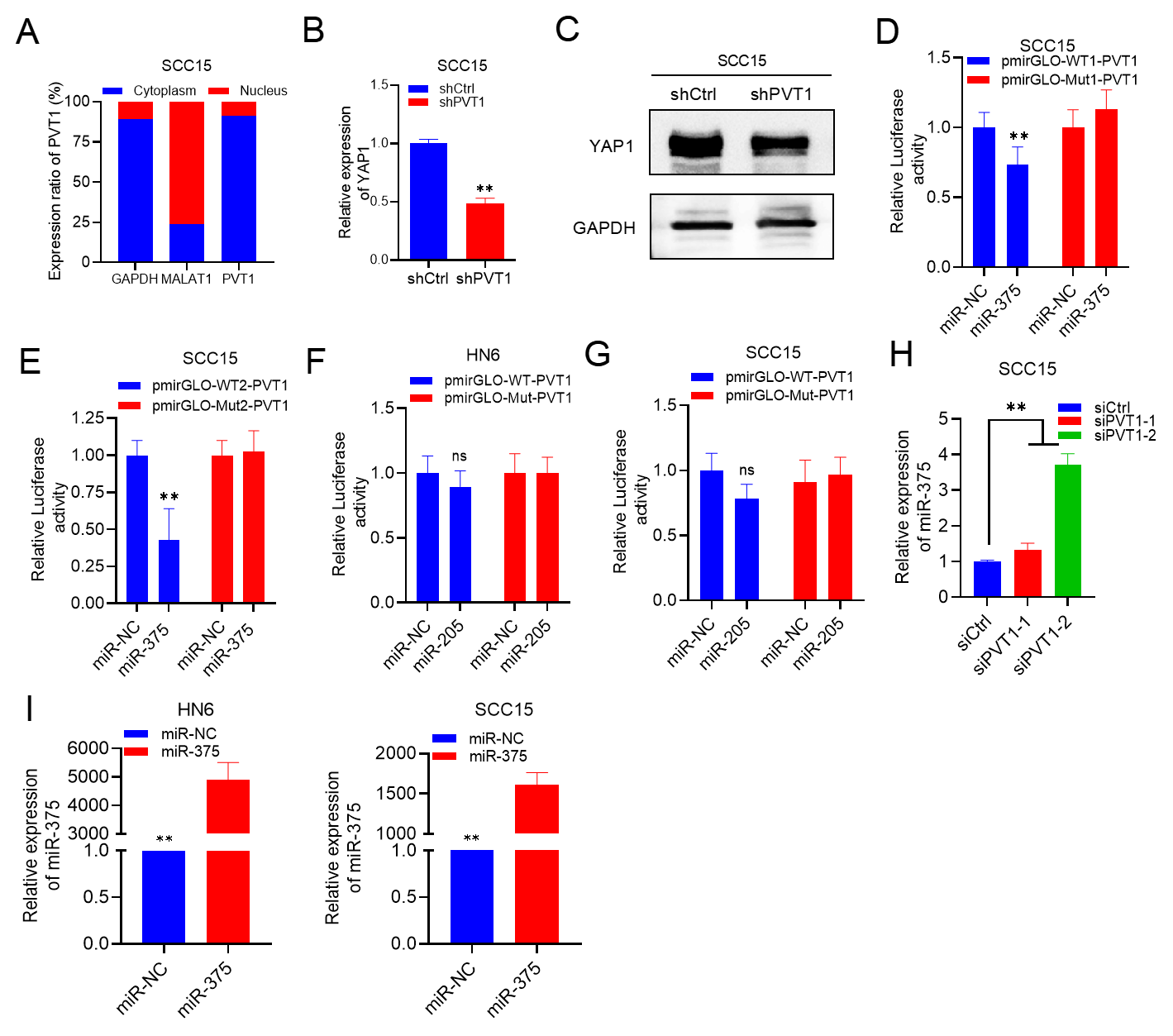


**Supplemental Fig. S4** **PVT1 and YAP1 are direct targets of miR-375.** **A** The subcellular localization of PVT1 by qRT-PCR analysis in SCC15 cells. MALAT1 and GAPDH transcripts were used as controls for the nuclear and cytoplasmic fractions. **B** qRT-PCR analysis of YAP1 expression in SCC15 cells with PVT1 KD. Data are shown as the mean ± SD. **p < 0.01 using an unpaired Student’s t-test. **C** Western blot analysis of YAP1 in SCC15 treated with PVT1 KD. **D-E** The relative luciferase activity in SCC15 transfected with pmirGLO-WT1-PVT1, pmirGLO-Mut1-PVT1, pmirGLO-WT2-PVT1, pmirGLO-Mut2-PVT1, and miR-375. Data are shown as the mean ± SD. **p < 0.01 using an unpaired Student’s t-test. **F-G** The relative luciferase activities of HN6 and SCC15 cells transfected with pmirGLO-WT-PVT1, pmirGLO-Mut-PVT1, and miR-205 mimics (miR-205). Data are shown as the mean ± SD. ns, p > 0.05 using an unpaired Student’s t-test. **H** qRT-PCR assessment of miR-375 expression in SCC15 cells transfected with siPVT1-1/2. Data are shown as the mean ± SD. **p < 0.01 using an unpaired Student’s t-test. **I** qRT-PCR assessment of miR-375 expression in HN6 and SCC15 cells transfected with miR-375. Data are shown as the mean ± SD. **p < 0.01 using an unpaired Student’s t-test.


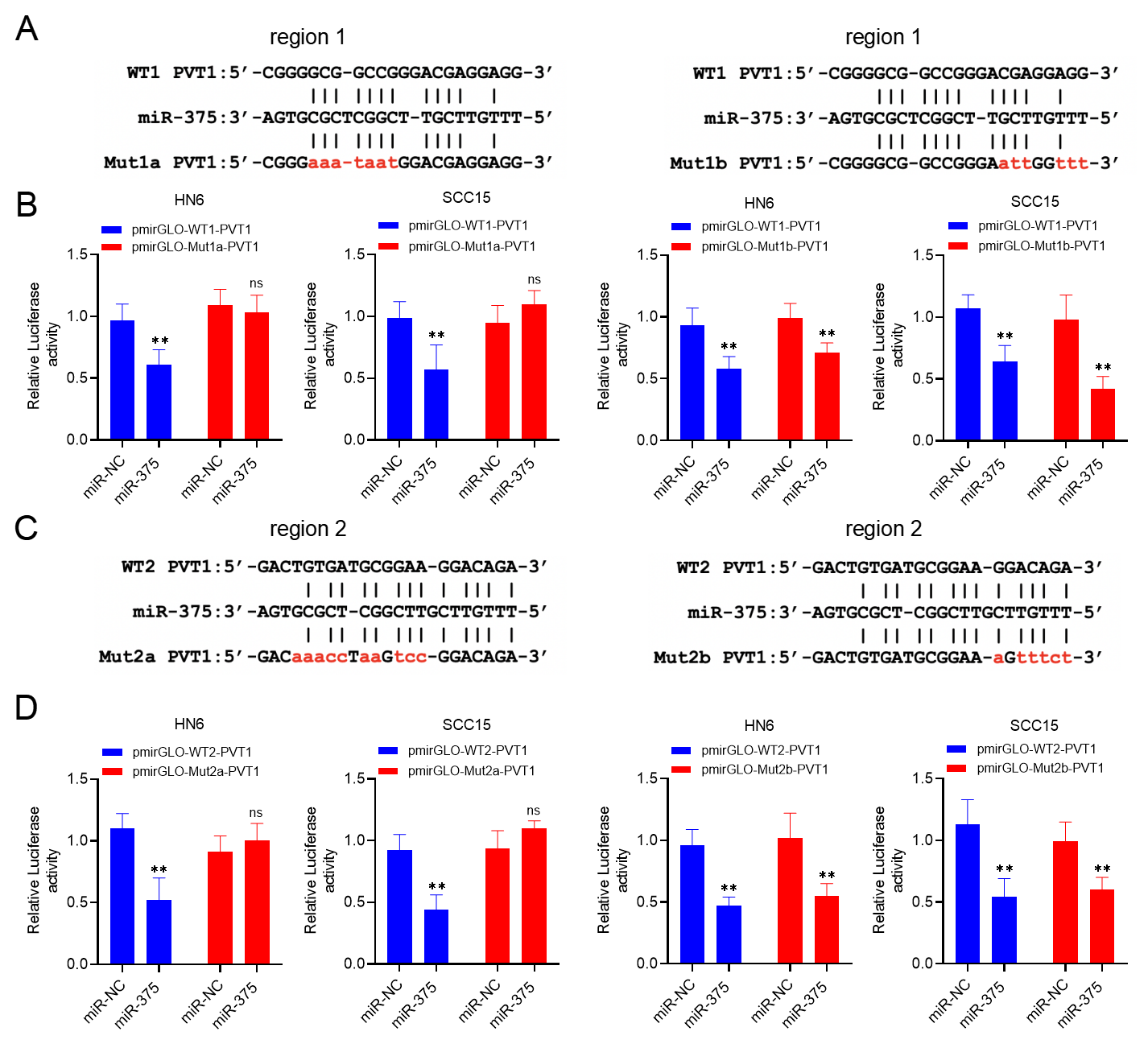


**Supplemental Fig. S5 PVT1 serves as a sponge for miR-375 in HNSCC cells. A** Bioinformatic analysis of miR-375 binding to region 1 of PVT1. **B** The relative luciferase activity in HN6 and SCC15 cells transfected with pmirGLO-WT1-PVT1, pmirGLO-Mut1a-PVT1, pmirGLO-Mut1b-PVT1, and miR-375 mimics (miR-375). Data are shown as the mean ± SD. ns, p > 0.05, **p < 0.01 by an unpaired Student’s t-test. **C** Bioinformatic analysis of miR-375 binding to region 2 of PVT1. **D** The relative luciferase activity in HN6 and SCC15 cells transfected with pmirGLO-WT2a-PVT1, pmirGLO-WT2b-PVT1, pmirGLO-Mut2-PVT1 and miR-375. Data are shown as the mean ± SD. ns, p > 0.05 and **p < 0.01 using an unpaired Student’s t-test.


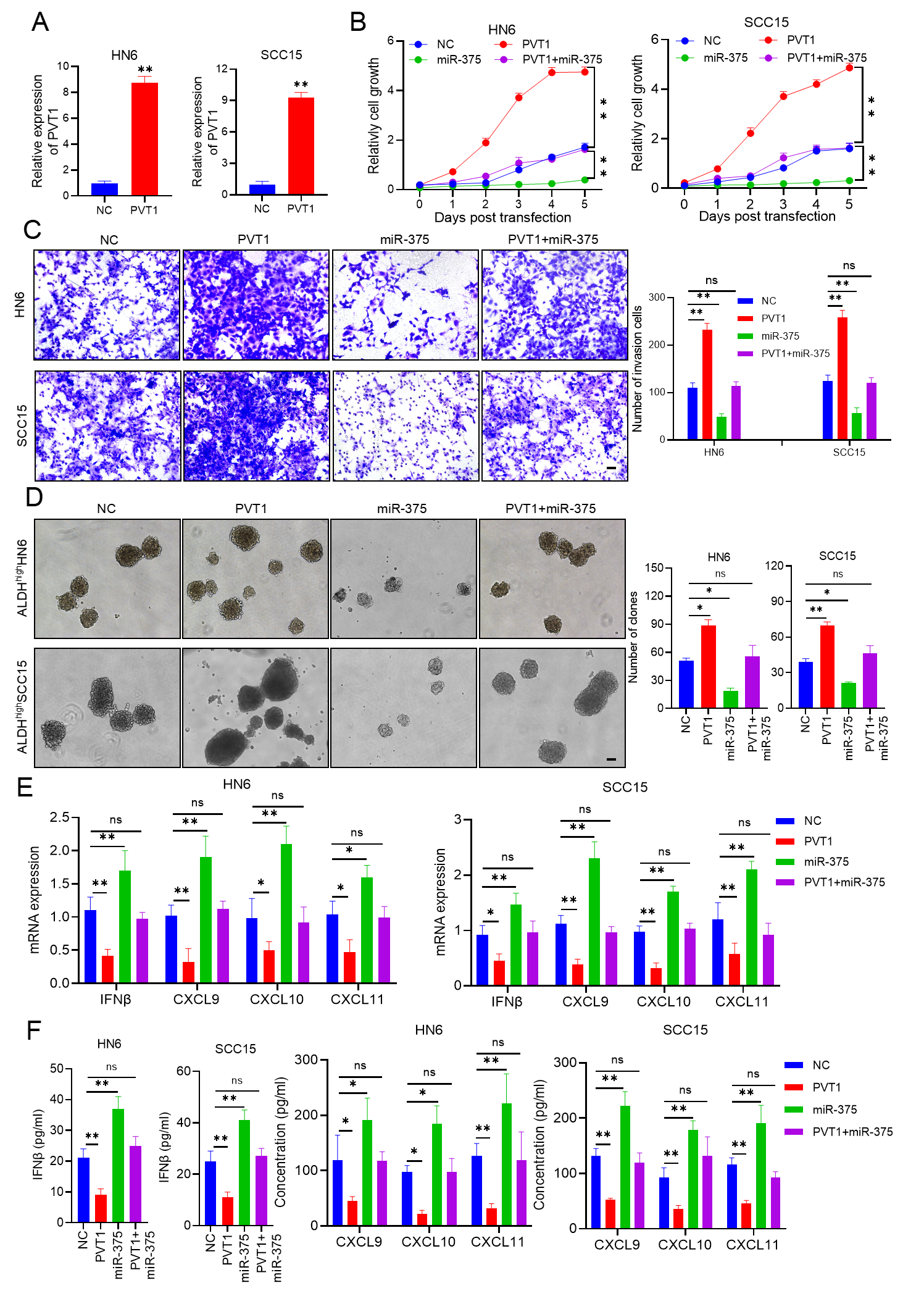


**Supplemental Fig. S6 MiR-375 mimics reverse the oncogenic function of PVT1.**

**A** qRT-PCR assessment of PVT1 expression in HN6 and SCC15 cells transfected with the lentivirus pQLL-lncPVT1 (PVT1). Data are shown as the mean ± SD. **p < 0.01 using an unpaired Student’s t-test. **B** The impact of co-transfection with PVT1 and miR-375 mimics on HN6 and SCC15 cells proliferation. Data are shown as the mean ± SD. **p < 0.01 using one-way ANOVA. **C** The impact of co-transfection with PVT1 and miR-375 mimics on HN6 and SCC15 cell invasion after 24 h. Scale bar: 100 μm. Data are shown as the mean ± SD. ns, p > 0.05 and **p < 0.01 using one-way ANOVA. **D** Image and numbers of tumorspheres formed by ALDH^high^ HNSCC cells co-transfected with PVT1 and miR-375 mimics. Scale bar: 100 μm. Data are shown as the mean ± SD. ns, p > 0.05, *p < 0.05, **p < 0.01 using one-way ANOVA. **E** qRT-PCR assessment of the expression levels of IFNβ, CXCL9, CXCL10, and CXCL11 in HN6 and SCC15 cells co-transfected with PVT1 and miR-375 mimics. Data are shown as the mean ± SD. ns, p > 0.05, *p < 0.05, **p < 0.01 using one-way ANOVA. **F** ELISA analysis of the protein levels of IFNβ, CXCL9, CXCL10, and CXCL11 secreted by HN6 and SCC15 cells co-transfected with PVT1 and miR-375 mimics. Means ± SD are shown. ns, p > 0.05, *p < 0.05, **p < 0.01 using one-way ANOVA.

**Supplemental Table S1. Sequences of qRT-PCR primers, other primers, and siRNAs used in the present study**

| Target gene | Forward primer (5′-3′) | Reverse primer (3′-5′) |
| --- | --- | --- |
| GAPDH | AACGGGAAGCTTGTCATCAA | TGGACTCCACGACGTACTCA |
| CD24 | TGAAGAACATGTGAGAGGTTTGAC | GAAAACTGAATCTCCATTCCACAA |
| BMI1 | TGCTTTGGTCGAACTTGGTG | TTTGCAGACTGGGGACAATG |
| SOX2 | GCCGAGTGGAAACTTTTGTCG | GGCAGCGTGTACTTATCCTTCT |
| ALDH1 | ACTTACCTGTCCTACTCA | CTTATCTCCTTCTTCTACCT |
| OCT4 | TCCCATGCATTCAAACTGAGG | CCTTTGTGTTCCCAATTCCTTCC |
| LASTR | GTGGGTGAAGTCCTGGTT | GGCTGAAGGGTTTAGATG |
| lnc-LAMC2 | CACGAACTTGTGGTTACTTGCTCAC | ATCCAAACCAACATCCACCCC |
| lnc-ZNF33B | CCTCAGTTTGGGAATTCAGTG | AAGCAAGCAGGCGAAGAA |
| PVT1 | TTCAGCACTCTGGACGGACTT | TATGGCATGGGCAGGGTAG |
| MIR205HG | GACCGTTGTTAGCACGCCTT | CACGTATCGGTCCGTGTTGG |
| ENTPD1-AS1 | GCAGGTTTACTCCACATACTGTGTT | ACCACAAGTTTTAGAGGTTTAAGCC |
| IFNβ | GCCATCAGTCACTTAAACAGC | GAAACTGAAGATCTCCTAGCCT |
| CXCL9 | GTGGTGTTCTTTTCCTCTTGGG | ACAGCGACCCTTTCTCACTAC |
| CXCL10 | GCAAGCCAATTTTGTCCACG | ACATTTCCTTGCTAACTGCTTTCAG |
| CXCL11 | CAGAATTCCACTGCCCAAAGG | GTAAACTCCGATGGTAACCAGCC |
| MALAT1 | GTTCTGATCCCGCTGCTATT | TCCTCAACACTCAGCCTTTATC |
| YAP1 | TAGCCCTGCGTAGCCAGTTA | TCATGCTTAGTCCACTGTCTGT |
| miR-375-RT | GTCGTATCCAGTGCAGGGTCCGAGGTATTCGCACTGGATACGACTCACGC | |
| miR-375-qPCR | GTG CAG GGT CCG AGGT | AGC CGT TTG TTC GTT CGG CT |
| Target gene | siRNA sequence (5′-3′) |  |
| siPVT1-1 | CAGCTTCAACCCATTACGATT |  |
| siPVT1-2 | GCCATCATGATGGTACTTTAA |  |
| shPVT1 | CCUGAUGGAUUUACAGUGATT |  |
