# Supplementary material for: PVT1 inhibition stimulates anti-tumor immunity, prevents metastasis, and depletes cancer stem cells in squamous cell carcinoma
Source: Cell Death Dis. 2023 Mar 9;14(3):187. doi: 10.1038/s41419-023-05710-6 (PMC9998619; doi:10.1038/s41419-023-05710-6)
Supplement: Supplementary file 2 — PVT1 Supplemental file_Uncropped WB [file 41419_2023_5710_MOESM2_ESM.docx]

Full and uncropped western blot for Figure 3E

HN6 BMI1, HN6 GAPDH, SCC15 BMI1, SCC15 GAPDH are on the figure.

HN6 GAPDH

HN6 BMI1

SCC15 BMI1

SCC15 GAPDH

Full and uncropped western blot for Figure 7B

HN6 pH2A.X, HN6 GAPDH, SCC15 pH2A.X, SCC15 GAPDH are on the figure.

HN6. GAPDH

HN6 pH2A.X

SCC15 GAPDH

SCC15 pH2A.X

Full and uncropped western blot for Figure 8C

HN6 YAP1, HN6 GAPDH are on the figure.

HN6 GAPDH

HN6 YAP1

Full and uncropped western blot for Supplemental Figure S4C

SCC15 YAP1, SCC15 GAPDH are on the figure.

SCC15 YAP1

SCC15 GAPDH

Full and uncropped western blot for Figure 8L

HN6 YAP1, HN6 GAPDH, SCC15 YAP1, SCC15 GAPDH are on the figure.

HN6 GAPDH

HN6 YAP1

SCC15 YAP1

SCC15 GAPDH

Full and uncropped western blot for Supplemental Fig. S1A

HN6 CD24, HN6 BMI1, HN6 SOX2, HN6 ALDH1, HN6 OCT4, HN6 GAPDH are on the figure.

HN6 BMI1

HN6 CD24

HN6 ALDH1

HN6 SOX2

HN6 GAPDH

HN6 OCT4

Full and uncropped western blot for Supplemental Fig. S1B

HN6 CD24, HN6 SOX2, HN6 ALDH1, HN6 OCT4, HN6 GAPDH are on the figure.

SCC15 CD24, SCC15 SOX2, SCC15 ALDH1, SCC15 OCT4, SCC15 GAPDH are on the figure.

SCC15 CD24

HN6 CD24

SCC15 SOX2

HN6 SOX2

HN6 ALDH1

SCC15 ALDH1

SCC15 OCT4

HN6 OCT4

SCC15 GAPDH

HN6 GAPDH
